# Supplementary material for: MetaRibo-Seq measures translation in microbiomes
Source: Nat Commun. 2020 Jun 29;11:3268. doi: 10.1038/s41467-020-17081-z (PMC7324362; doi:10.1038/s41467-020-17081-z)
Supplement: Supplementary file 10 — Supplementary Data 7 [file 41467_2020_17081_MOESM10_ESM.zip › File2/Confidence_High_Taxonomy/115647_out.krona.html]

Javascript must be enabled to view this page.

members
magnitude
magnitudeUnassigned
count
unassigned
taxon
rank

115647\_out

9

9
superkingdom
2

6
1239
phylum

2

SRS019496\_contig\_number\_contig-100\_6247.53973SRS078176\_contig\_number\_contig-100\_836.120769
species
1263024

4
91061
class

4
1385
order

186817
family
1

genus
1386
1

1

SRS076804\_contig\_number\_20861
species
859143

3
90964
family

1279
genus
3

species
1262969
3

SRS049995\_contig\_number\_35343SRS098644\_contig\_number\_47779SRS142503\_contig\_number\_50024

phylum
544448
3

3
31969
class

186329
order
3

3

SRS053356\_contig\_number\_31119SRS1041039\_contig\_number\_8510SRS104311\_contig\_number\_45999
2053494
species
